# Associations Between Lead and Cadmium Exposure and Subclinical Cardiovascular Disease in U.S. Adults

**DOI:** 10.1007/s12012-024-09955-1

**Published:** 2025-01-28

**Authors:** Lin Liu, Aimin Xu, Bernard M. Y. Cheung

**Affiliations:** 1 Department of Medicine, School of Clinical Medicine, The University of Hong Kong, Pokfulam, Hong Kong China; 2 State Key Laboratory of Pharmaceutical Biotechnology, The University of Hong Kong, Pokfulam, Hong Kong China; 3 Institute of Cardiovascular Science and Medicine, The University of Hong Kong, Pokfulam, Hong Kong China; 4 Department of Medicine, Queen Mary Hospital, University of Hong Kong, Pokfulam, Hong Kong China

**Keywords:** Heavy metals, Lead, Cadmium, NT-proBNP, Troponin

## Abstract

The impact of lead and cadmium exposure on subclinical cardiovascular disease (CVD), indicated by elevated high-sensitivity cardiac troponin (hs-cTnT) and N-terminal pro b-type natriuretic peptide (NT-proBNP) remains uncertain. We analyzed data from participants aged 20 and older, without overt CVD, in the National Health and Nutrition Examination Survey (NHANES; 1999–2004). Elevated lead and cadmium levels were defined as 3.5 μg/dL and 1.0 μg/L (inductively coupled plasma mass spectrometry) and 3.8 μg/dL and 0.9 μg/L (atomic absorption spectrometry), respectively. Elevated hs-cTnT was ≥ 19 ng/L, and elevated NT-proBNP was ≥ 125 pg/mL. Multivariate logistic regression estimated the odds ratios (OR) and 95% confidence intervals (CI) for elevated biomarkers. Among 10,197 participants (mean age 48.8 years; 50.3% female), 5.3% had elevated hs-cTnT and 19.4% had elevated NT-proBNP. Elevated blood lead was associated with increased ORs for elevated hs-cTnT (OR 1.45, 95% CI 1.15–1.84) and NT-proBNP (OR 1.66, 95% CI 1.40–1.97). The corresponding ORs (95% CI) for elevated blood cadmium were 1.33 (1.02, 1.74) and 1.39 (1.18, 1.65). The effect of elevated blood lead on NT-proBNP was particularly pronounced among non-Hispanic Blacks (OR [95% CI], 3.26 [2.24, 4.74]) compared to Mexican Americans (1.46 [0.99, 2.17]) and non-Hispanic Whites (1.31 [1.02, 1.68]) and was stronger in individuals with impaired kidney function (OR [95% CI], 2.31 [1.43, 3.75]) compared to those with normal kidney function (1.44 [1.18, 1.75]). This study first reveals the association between lead and cadmium exposure and subclinical CVD, underscoring the need for targeted preventive measures to reduce cardiovascular risk and improve health outcomes.

**Supplementary Information:**

The online version contains supplementary material available at 10.1007/s12012-024-09955-1.

## Introduction

The global burden of cardiovascular disease (CVD) remains a significant public health challenge, underscoring the necessity for a comprehensive understanding of both traditional and emerging risk factors [1]. While the effects of recognized contributors such as hypertension, diabetes, hyperlipidemia have been thoroughly studied, the role of environmental toxins, particularly heavy metals like lead and cadmium, is gaining recognition. These metals are associated with persistent adverse impacts on the cardiovascular system, including hypertension, arrhythmias, and atherosclerosis [2]. Lead and cadmium, non-essential and pervasive in the environment, enter human systems via air, water, soil, and food, and their extensive industrial and public use compounds their prevalence. These metals have half-lives spanning decades, posing a long-term public health concern [3].

Recent research has pivoted towards subclinical CVD, defined as pathological changes that manifest prior to clinical symptoms, thus offering a window for early intervention. Novel biomarkers, such as Growth Differentiation Factor-15 (GDF-15), soluble suppression of tumorigenicity 2 (sST2), high-sensitivity cardiac troponin T (hs-cTnT), and N-terminal pro-B-type natriuretic peptide (NT-proBNP), have emerged as valuable tools for identifying early cardiac damage [4]. Among these, hs-cTnT and NT-proBNP have gained widespread clinical use and are recommended by the American Heart Association (AHA) for early screening of subclinical CVD [5]. Hs-cTnT is a highly sensitive and specific biomarker for detecting myocardial injury (MI), while NT-proBNP is a reliable indicator of heart failure (HF), two of the most severe manifestations of CVD. Interestingly, elevated levels of hs-cTnT and NT-proBNP have also been observed in asymptomatic individuals and are associated with an increased risk of adverse health outcomes [6, 7]. Emerging evidence suggests that heavy metals, such as lead and cadmium, may play a significant role in the pathogenesis of CVD by inducing inflammatory responses, endothelial dysfunction, vascular impairment, and direct cellular damage leading to apoptosis [8]. These mechanisms overlap with the pathways contributing to MI and HF. However, limited research has specifically explored the associations between lead and cadmium exposure and subclinical elevations in hs-cTnT and NT-proBNP. Addressing this gap is critical for understanding the cardiovascular risks posed by environmental heavy metal exposure and for identifying at-risk populations who may benefit from targeted interventions.

This study aims to investigate the association between blood levels of lead and cadmium and subclinical cardiovascular disease, as evidenced by elevated hs-cTnT and NT-proBNP, in a diverse cohort of U.S. adults. By delineating the connection between these environmental exposures and early cardiovascular alterations, our research endeavors to guide targeted preventive strategies and bolster public health policies.

## Method

### Study Population

Data for this study were obtained from the National Health and Nutrition Examination Survey (NHANES), a program designed to assess the health and nutritional status of individuals in the United States. Details of the analytical guidelines have been published elsewhere [9]. For this study, we analyzed NHANES data collected from 1999 to 2004, initially comprising 31,126 participants. After excluding individuals aged 19 years or younger (N = 15,794), those lacking blood lead or cadmium measurements (N = 1828), those missing NT-proBNP or hs-cTnT measurements (N = 1238), pregnant participants (N = 660), and participants with known cardiovascular diseases (CVD) including coronary artery disease, angina, myocardial infarction, heart failure, and stroke (N = 699), the final sample included 10,197 individuals. A study flowchart is depicted in Fig. S1. NHANES was conducted by the National Center for Health Statistics of the Centers for Disease Control and Prevention (CDC), with approval from the institutional review board of the National Center for Health Statistics. All participants provided written informed consent.

### Measurement of Blood Lead and Cadmium

Blood Samples were Collected at the Mobile Examination Centers (MEC). For the 1999–2002 cycles, blood levels of cadmium and lead were measured using a PerkinElmer Model SIMAA 6000 atomic absorption spectrometer (AAS) with Zeeman background correction. Detection limits were established at 0.3 µg/dL for lead and 0.3 µg/L for cadmium. For the 2003–2004 cycles, inductively coupled plasma mass spectrometry (ICP-MS, PerkinElmer ELAN® ICP-DRC-MS System) was employed, with detection thresholds at 0.3 µg/dL for lead and 0.2 µg/L for cadmium [10]. Participants were categorized into quartiles based on their metal levels, with detailed cutoffs provided in Table S1. Additionally, participants were categorized as elevated or not elevated based on recommended cutoffs of 3.5 μg/dL for lead (n = 432 [12.4%]) and 1.0 μg/L for cadmium (n = 506 [14.5%]), as measured by ICP-MS [2]. The cutoffs for AAS-measured lead and cadmium have not been established; we chose cutoffs that corresponded to a similar percentage as ICP-MS results. Thus, the cutoff for lead is 3.8 μg/dL (n = 865 [12.4%]), and for cadmium is 0.9 μg/L (n = 1068 [14.5%]), measured by AAS.

### Measurement of hs-cTnT and NT-proBNP

Hs-cTnT and NT-proBNP levels were measured on stored serum samples at the University of Maryland between 2018 and 2020 [11]. The hs-cTnT assay was conducted using the Roche Cobas e601 analyzer with Elecsys reagents, showing a coefficient of variation between 2.0% and 3.1% and a detection limit of 3 ng/L. The NT-proBNP assay demonstrated a coefficient of variation from 2.7% to 3.1%, with detection thresholds ranging from 5 pg/mL to 35,000 pg/mL. According to the recommendations of the International Federation of Clinical Chemistry and Laboratory Medicine (IFCC), elevated biomarker levels are defined as hs-cTnT ≥ 19 ng/L and NT-proBNP ≥ 125 pg/mL [12, 13], criteria that were further validated by a previous study [11]. Additionally, the American Heart Association (AHA) has proposed sex-specific cutoffs for hs-cTnT (≥ 14 ng/L for women and ≥ 22 ng/L for men) [5]. To account for these differences, we also conducted sensitivity analyses using the sex-specific cutoffs.

### Other Study Variables

Demographic and health-related data collected included age, gender, race/ethnicity (self-reported as non-Hispanic White, non-Hispanic Black, Mexican–American, or Other), education, and smoking status (current smoker, former smoker, nonsmoker). Clinical measurements obtained were systolic blood pressures (SBP), diastolic blood pressures (DBP), body mass index (BMI), total cholesterol (TC), hemoglobin A1c (HbA1c), and serum creatinine (Scr). Medication information included antihypertensive drug, antidiabetic drug, and statin were also collected. Hypertension was defined as a mean SBP ≥ 140 mmHg, a mean DBP ≥ 90 mmHg, taking antihypertensive drugs, or a self-reported history. Diabetes was defined as HbA1c ≥ 6.5%, taking antidiabetic drugs, or a self-reported history. Dyslipidemia was defined as total cholesterol ≥ 240 mg/dL, taking statins, or a self-reported history. The estimated glomerular filtration rate (eGFR) was calculated using the Modification of Diet in Renal Disease (MDRD) formula [14].

### Statistical Analysis

Continuous variables were presented as means ± standard deviation (SD) for normally distributed data, or medians with interquartile ranges [Q1, Q3] for skewed data. Differences among groups were assessed using the Kruskal–Wallis test for nonparametric data and one-way analysis of variance (ANOVA) for parametric data.

To assess the association between blood levels of lead and cadmium and elevated biomarkers of cardiac stress (hs-cTnT and NT-proBNP), logistic regression models were used. Odds ratios (ORs) and 95% confidence intervals (95% CIs) were computed to estimate the likelihood of elevated biomarker levels associated with lead and cadmium exposure. Two models were developed: Model 1 was unadjusted. Model 2 was adjusted for age, gender, race/ethnicity, smoking status, SBP, BMI, TC, HbA1c, eGFR, diabetic status, use of anti-diabetic drugs, hypertension, use of anti-hypertensive drugs, and statin use. Analysis was further stratified by quartiles of metal levels, using the lowest quartile as the reference group.

For continuous analysis, log-transformations (natural logarithm) were applied to both heavy metal levels and cardiac biomarkers to normalize their skewed distributions. The Pearson correlation was used to estimate the relationship between log-transformed metal levels and log-transformed cardiac biomarkers. The association between log-transformed metal levels and elevated cardiac biomarkers was modeled using restricted cubic splines, with knots placed at the 25th, 50th, and 75th percentiles. Measurements below the limit of detection (LOD) were excluded where appropriate.

Subgroup analyses were conducted across various demographic and clinical characteristics: age groups (< 65 vs. ≥ 65 years), Gender (female vs. male), race/ethnicity (non-Hispanic White, non-Hispanic Black, Mexican–American, Other), smoking status (current smoker, former smoker, nonsmoker), presence of hypertension (yes vs. no), diabetes status (yes vs. no), eGFR categories (≥ 60 vs. < 60 mL/min/1.73 m^2^). Interaction terms were tested in the logistic models, and P-values for interaction were adjusted using the Bonferroni method for multiple comparisons when applicable.

Survey weights were not applied to the main analyses for two reasons: the sample was highly selected from the study population [15], and the weights for metal and hs-cTnT/NT-proBNP measurements differed [16]. Specifically, heavy metal levels were measured in the Mobile Examination Center (MEC) between 1999 and 2004, and MEC exam weights were applied for these measurements. Hs-cTnT and NT-proBNP levels were measured on stored serum samples between 2018 and 2020, with subsample weights applied for these measurements. However, survey weights were applied to the logistic regression models using different weights mentioned above for sensitivity analyses.

Several sensitivity analyses were conducted to validate the robustness of our findings. First, participants were stratified into sex-specific quartiles for lead and cadmium, with cutoffs shown in Table S1. Second, alternative cutoffs were applied to define elevated hs-cTnT (≥ 14 ng/L for women and ≥ 22 ng/L for men) and elevated NT-proBNP (≥ 125 pg/mL for adults < 75 years and ≥ 450 pg/mL for adults ≥ 75 years) [11]. Third, survey weights were applied to logistic regression models, using different weights for metal levels and biomarker measurements.

All statistical analyses were conducted using R software, version 4.2.1 (R Foundation for Statistical Computing). A two-tailed P-value of less than 0.05 was considered indicative of statistical significance.

## Results

### Baseline Characteristic

Table 1 presents the baseline characteristics of the study population, comprising 10,393 participants (mean age ± SD: 48.8 ± 18.2 years; 50.3% female). Notable distinctions were observed in participants with elevated blood lead levels—they were typically older, predominantly male, Non-Hispanic Balck, and current smokers. These participants also exhibited lower educational levels, BMI, and eGFR, alongside higher SBP, TC, NT-proBNP, hs-cTnT, and a higher prevalence of hypertension compared to those with non-elevated lead levels. Participants with elevated blood cadmium levels were more likely to be male, Non-Hispanic Black, and current smokers, with lower educational levels and BMI, but higher NT-proBNP levels. Similar patterns across quartiles of blood lead and cadmium levels were detailed in supplementary tables (Tables S2, S3).

**Table 1:** Baseline Characteristic according to Blood Lead and Cadmium Levels

	Overall	Blood lead, μg/dL			Blood cadmium, μg/L		
		Not elevated	Elevated	P-value	Not elevated	Elevated	P-value
	10,197	8900	1297		8623	1574	
Age (years)	48.8 ± 18.2	47.7 ± 18.1	56.7 ± 17.5	< 0.001	48.8 ± 18.5	48.9 ± 17.1	0.82
Gender, %				< 0.001			0.03
Male	5063 (49.7)	4118 (46.3)	945 (72.9)		4240 (49.2)	823 (52.3)	
Female	5134 (50.3)	4782 (53.7)	352 (27.1)		4383 (50.8)	751 (47.7)	
Race/Ethnicity, %				< 0.001			< 0.001
Mexican–American	2367 (23.2)	2013 (22.6)	354 (27.3)		2117 (24.6)	250 (15.9)	
Non-Hispanic White	5115 (50.2)	4581 (51.5)	534 (41.2)		4282 (49.7)	833 (52.9)	
Non-Hispanic Black	1876 (18.4)	1556 (17.5)	320 (24.7)		1526 (17.7)	350 (22.2)	
Other	839 (8.2)	750 (8.4)	89 (6.9)		698 (8.1)	141 (9.0)	
Education, %				< 0.001			< 0.001
Less than high school	3161 (31.0)	2534 (28.5)	627 (48.3)		2566 (29.8)	595 (37.8)	
High school	2451 (24.0)	2147 (24.1)	304 (23.4)		1985 (23.0)	466 (29.6)	
College or higher	4585 (45.0)	4219 (47.4)	366 (28.2)		4072 (47.2)	513 (32.6)	
Smoking status, %				< 0.001			< 0.001
Current smoker	2656 (26.0)	2130 (23.9)	526 (40.6)		1405 (16.3)	1251 (79.5)	
Former smoker	2593 (25.4)	2212 (24.9)	381 (29.4)		2421 (28.1)	172 (10.9)	
Nonsmoker	4948 (48.5)	4558 (51.2)	390 (30.1)		4797 (55.6)	151 (9.6)	
SBP (mmHg)	126.3 ± 20.2	125.3 ± 19.7	132.7 ± 22.2	< 0.001	126.3 ± 20.3	126.2 ± 19.7	0.803
DBP (mmHg)	71.4 ± 13.1	71.4 ± 12.7	71.8 ± 15.5	0.333	71.5 ± 13.0	71.0 ± 13.6	0.124
BMI (kg/m2)	28.2 ± 6.1	28.3 ± 6.2	27.0 ± 5.14	< 0.001	28.4 ± 6.1	26.8 ± 5.8	< 0.001
TC (mg/dL)	202.8 ± 41.4	202.5 ± 41.6	204.9 ± 39.7	0.048	202.5 ± 41.5	204.1 ± 40.6	0.152
HbA1c (%)	5.5 ± 1.0	5.6 ± 1.0	5.6 ± 0.8	0.695	5.6 ± 1.0	5.6 ± 0.9	0.426
eGFR (mL/min/1.73 m2)	92.8 ± 31.4	93.3 ± 31.4	88.9 ± 30.9	< 0.001	92.8 ± 32.0	92.3 ± 27.3	0.575
NT-proBNP (pg/mL)	45.6 [21.9, 98.6]	44.2 [21.3, 93.4]	60.9 [26.6, 166.0]	< 0.001	45.0 [21.4, 97.0]	50.2 [24.0, 108.2]	< 0.001
Hs-cTnT (ng/L)	5.3 [3.7, 8.2]	5.1 [3.6, 7.8]	7.0 [4.8, 11.9]	< 0.001	5.3 [3.7, 8.2]	5.4 [3.8, 8.6]	0.113
Hypertension, %	3146 (30.9)	2652 (29.8)	494 (38.1)	< 0.001	2665 (30.9)	481 (30.6)	0.807
Anti-hypertensive drug, %	1854 (18.2)	1565 (17.6)	289 (22.3)	< 0.001	1593 (18.5)	261 (16.6)	0.079
Diabetes mellitus, %	1082 (10.6)	952 (10.7)	130 (10.0)	0.492	936 (10.9)	146 (9.3)	0.068
Anti-diabetic drug, %	662 (6.5)	602 (6.8)	60 (4.6)	0.004	584 (6.8)	78 (5.0)	0.008
Statins, %	757 (7.4)	671 (7.5)	86 (6.6)	0.267	669 (7.8)	88 (5.6)	0.003
Elevated NT-proBNP, %	1978 (19.4)	1580 (17.8)	398 (30.7)	< 0.001	1638 (19.0)	340 (21.6)	0.018
Elevated hs-cTnT, %	544 (5.3)	392 (4.4)	152 (11.7)	< 0.001	452 (5.2)	92 (5.8)	0.358

Continuous variables are expressed as mean ± standard deviation or as median [IQR]

Categorical variables are expressed as number (percent)

Elevated blood lead was defined as blood lead concentration > 3.5 μg/dL for inductively coupled plasma mass spectrometry and > 3.8 μg/dL for atomic absorption spectrometry

Elevated blood cadmium was defined as blood cadmium concentration > 1.0 μg/L for inductively coupled plasma mass spectrometry and > 0.9 μg/L for atomic absorption spectrometry

Elevated NT-proBNP was defined as blood NT-proBNP ≥ 125 pg/ml. Elevated hs-cTnT was defined as blood hs-cTnT ≥ 19 ng/L

*SBP* systolic blood pressure, *DBP* diastolic blood pressure, *BMI* body mass index, *TC* total cholesterol, *HbA1c* hemoglobin A1c, *eGFR* estimated glomerular rate, *Hs-cTnT* high sensitivity cardiac troponin, *NT-proBNP* N-terminal pro b-type natriuretic peptide

### Association Between Blood Lead and Cadmium with hs-cTnT and NT-proBNP

**Fig. 1:**
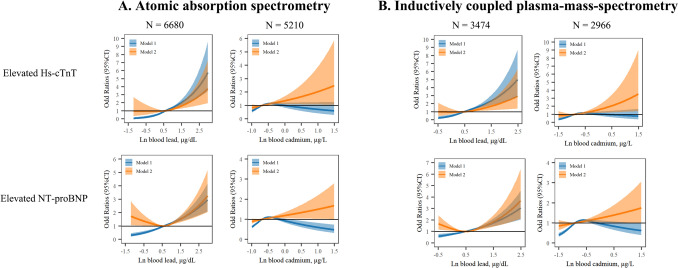
Odd Ratios (95% Confidence interval) of Elevated Hs-cTnT and NT-proBNP by Blood Lead and Cadmium. Hs-cTnT, high sensitivity cardiac troponin, NT-proBNP, N-terminal pro b-type natriuretic peptide. Atomic absorption spectrometry was used in NHANES, 1999–2002, inductively coupled plasma mass spectrometry was used in NHANES, 2003–2004. Elevated NT-proBNP was defined as blood NT-proBNP ≥ 125 pg/ml. Elevated hs-cTnT was defined as blood hs-cTnT ≥ 19 ng/L. Restrict cubic spline with knots set at the 25th, 50th, and 75th percentiles were used to fit the models. *Model 1* crude model. *Model 2* adjusted for age, gender, race/ethnicity, smoking status, systolic blood pressure, body mass index, total cholesterol, hemoglobin A1c, estimated glomerular rate, diabetes, anti-diabetic drug, hypertension, anti-hypertensive drug, and statin.

The Pearson correlations of blood lead and cadmium with hs-cTnT and NT-proBNP are shown in Fig. S1. Ln-transformed blood lead was positively correlated with Ln-transformed hs-cTnT and NT-proBNP, regardless of the laboratory method used. Similar correlations were observed between blood cadmium and Ln-transformed hs-cTnT and NT-proBNP. As shown in Table 2, higher blood lead and cadmium levels correlated with an increased likelihood of elevated NT-proBNP and hs-cTnT levels. Specifically, after adjusting for confounders, elevated lead levels resulted in ORs of 1.45 (95% CI: 1.15, 1.84) for hs-cTnT and 1.66 (95% CI: 1.40, 1.97) for NT-proBNP. Elevated cadmium levels showed ORs of 1.33 (95% CI: 1.02, 1.74) for hs-cTnT and 1.39 (95% CI: 1.18, 1.65) for NT-proBNP. These associations were consistent across quartiles of blood lead and cadmium levels. The relationships between log-transformed blood lead and cadmium with elevated biomarkers were largely linear in adjusted models, as illustrated in Fig. 1, regardless of the laboratory method used.

**Table 2:** Odd Ratios (95% Confidence interval) of Elevated Hs-cTnT and NT-proBNP by Blood Lead and Cadmium

	Elevated Hs-cTnT (N = 544)			Elevated NT-proBNP (N = 1978)		
	Cases/total	Model 1	Model 2	Cases/total	Model 1	Model 2
Blood lead						
Quartile 1	48/2550	Ref	Ref	313/2550	Ref	Ref
Quartile 2	92/2549	1.95 (1.37, 2.78)	1.00 (0.68, 1.47)	445/2549	1.51 (1.29, 1.77)	0.98 (0.81, 1.19)
Quartile 3	142/2549	3.08 (2.21, 4.29)	0.99 (0.68, 1.44)	530/2549	1.88 (1.61, 2.18)	0.91 (0.74, 1.10)
Quartile 4	262/2549	5.97 (4.37, 8.17)	1.46 (1.02, 2.11)	690/2549	2.65 (2.29, 3.07)	1.25 (1.02, 1.53)
Not elevated	392/8900	Ref	Ref	1580/8900	Ref	Ref
Elevated	152/1297	2.88 (2.37, 3.51)	1.45 (1.15, 1.84)	398/1297	2.05 (1.80, 2.34)	1.66 (1.40, 1.97)
Blood cadmium						
Quartile 1	82/2550	Ref	Ref	254/2550	Ref	Ref
Quartile 2	99/2549	1.22 (0.90, 1.64)	0.83 (0.60, 1.16)	436/2549	1.87 (1.58, 2.20)	1.13 (0.92, 1.37)
Quartile 3	181/2549	2.30 (1.76, 3.00)	1.06 (0.78, 1.44)	669/2549	3.22 (2.75, 3.76)	1.27 (1.05, 1.55)
Quartile 4	182/2549	2.31 (1.77, 3.02)	1.52 (1.12, 2.06)	619/2549	2.90 (2.48, 3.40)	1.61 (1.33, 1.96)
Not elevated	452/8623	Ref	Ref	1638/8623	Ref	Ref
Elevated	92/1574	1.12 (0.89, 1.41)	1.33 (1.02, 1.74)	340/1574	1.17 (1.03, 1.34)	1.39 (1.18, 1.65)

Hs-cTnT, high sensitivity cardiac troponin, NT-proBNP, N-terminal pro b-type natriuretic peptide

Elevated blood lead was defined as blood lead concentration > 3.5 μg/dL for inductively coupled plasma mass spectrometry and > 3.8 μg/dL for atomic absorption spectrometry

Elevated blood cadmium was defined as blood cadmium concentration > 1.0 μg/L for inductively coupled plasma mass spectrometry and > 0.9 μg/L for atomic absorption spectrometry

Elevated NT-proBNP was defined as blood NT-proBNP ≥ 125 pg/ml. Elevated hs-cTnT was defined as blood hs-cTnT ≥ 19 ng/L

Model 1, crude model, Model 2, adjusted for age, gender, race/ethnicity, smoking status, systolic blood pressure, body mass index, total cholesterol, hemoglobin A1c, estimated glomerular rate, diabetes, anti-diabetic drug, hypertension, anti-hypertensive drug, and statin

### Subgroup Analysis

**Fig. 2:**
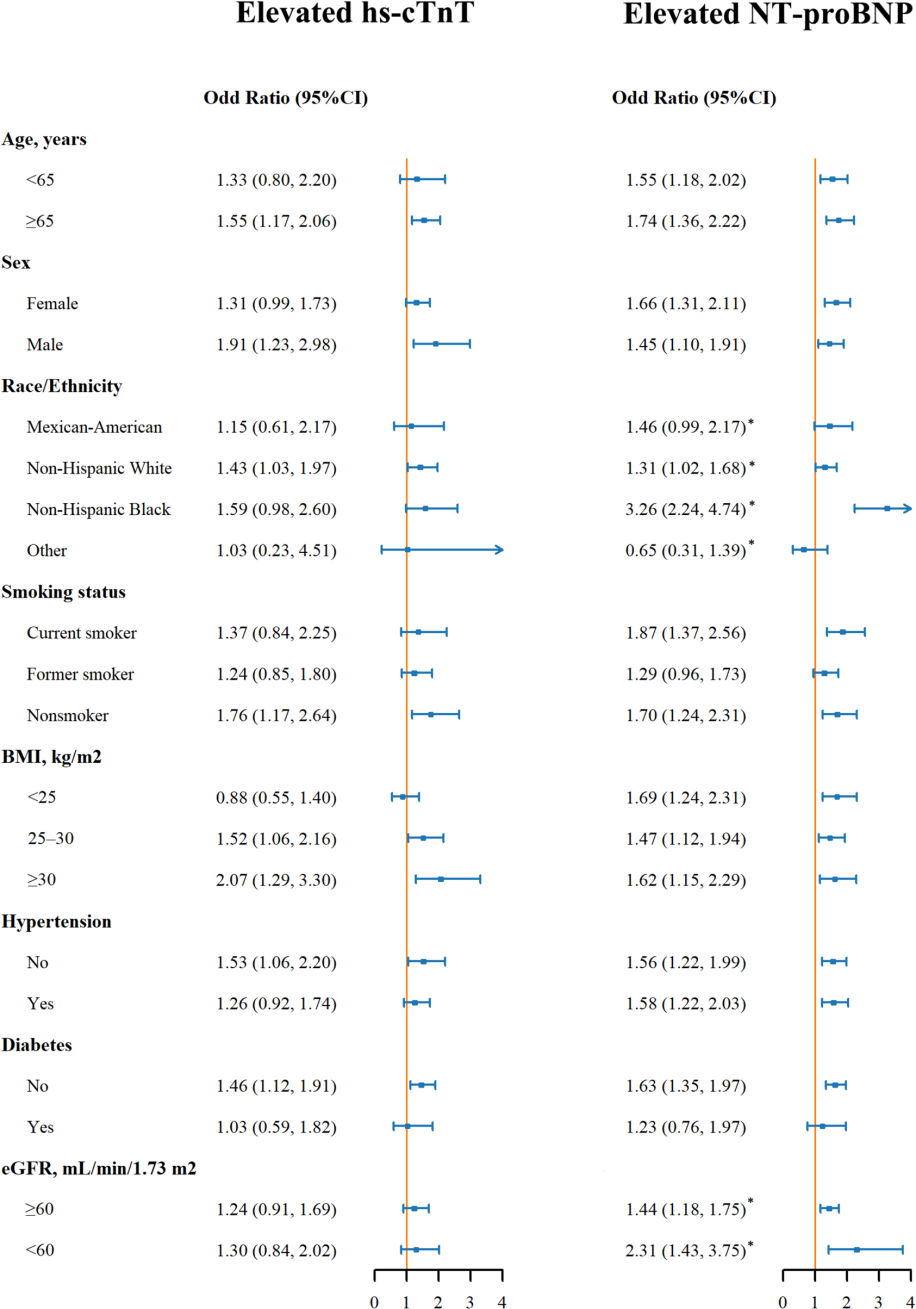
Odd Ratios (95% Confidence interval) of Elevated Hs-cTnT and NT-proBNP by Blood Lead in Subgroups. Odd ratios for elevated lead compared to not elevated were presented. Hs-cTnT, high sensitivity cardiac troponin, NT-proBNP, N-terminal pro b-type natriuretic peptide, BMI, body mass index, eGFR, estimated glomerular rate. Elevated NT-proBNP was defined as blood NT-proBNP ≥ 125 pg/ml. Elevated hs-cTnT was defined as blood hs-cTnT ≥ 19 ng/L. Models were adjusted for age, gender, race/ethnicity, smoking status, systolic blood pressure, body mass index, total cholesterol, hemoglobin A1c, estimated glomerular rate, diabetes, anti-diabetic drug, hypertension, anti-hypertensive drug, and statin. *P value for the interaction term was significant at Bonferroni corrected p value (0.008 for race/ethnicity and 0.05 for eGFR). P-values for Pairwise comparison: Non-Hispanic Black vs. Mexican American, 0.006, Non-Hispanic Black vs. Non-Hispanic White, < 0.001, Non-Hispanic Black vs. Other, < 0.001

**Fig. 3:**
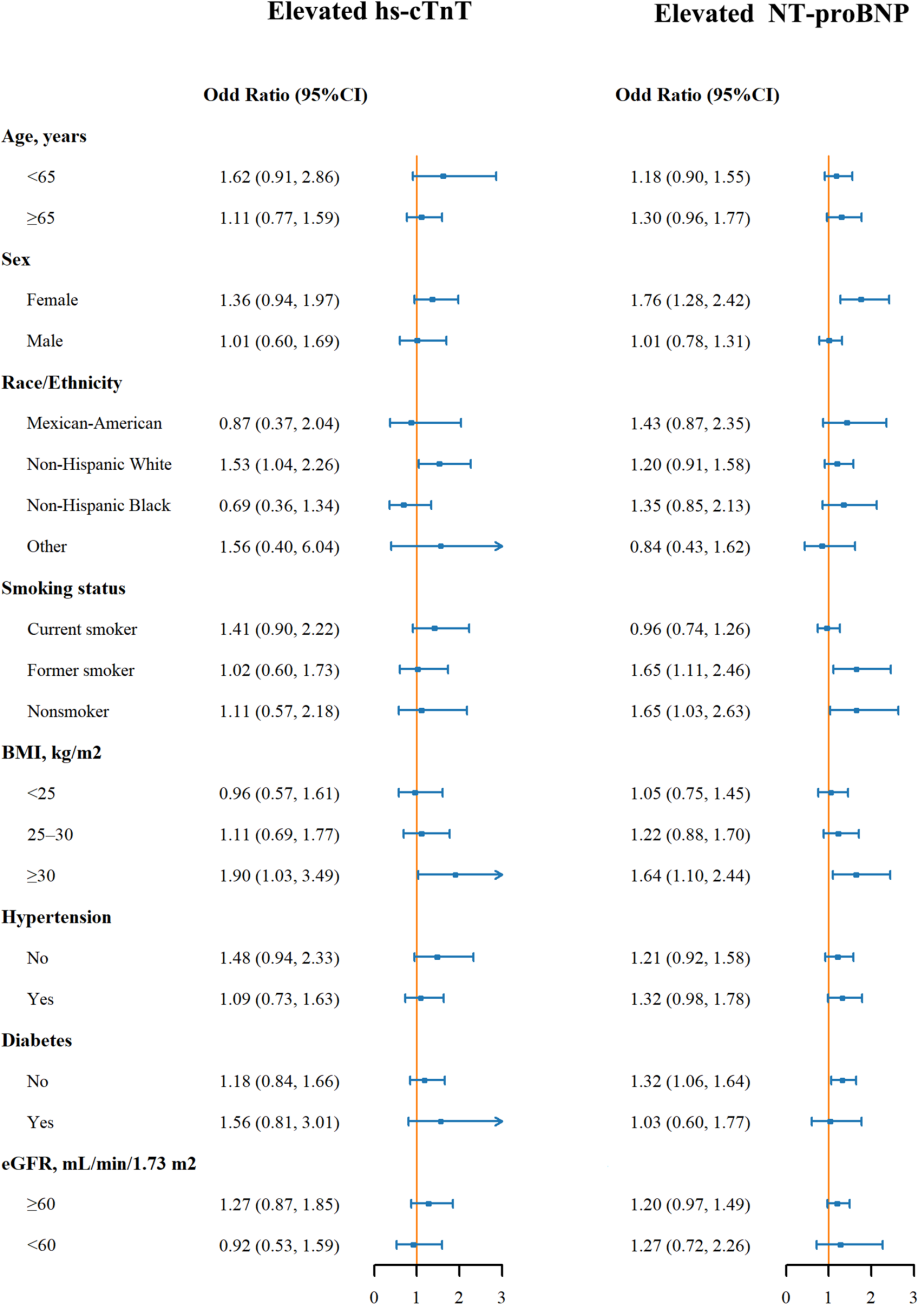
Odd Ratio (95% Confidence interval) of Elevated Hs-cTnT and NT-proBNP by Blood Cadmium in Subgroups. Odd ratios for elevated cadmium compared to not elevated were presented. Hs-cTnT, high sensitivity cardiac troponin, NT-proBNP, N-terminal pro b-type natriuretic peptide, BMI, body mass index, eGFR, estimated glomerular rate. Elevated NT-proBNP was defined as blood NT-proBNP ≥ 125 pg/ml. Elevated hs-cTnT was defined as blood hs-cTnT ≥ 19 ng/L. Models were adjusted for age, gender, race/ethnicity, smoking status, systolic blood pressure, body mass index, total cholesterol, hemoglobin A1c, estimated glomerular rate, diabetes, anti-diabetic drug, hypertension, anti-hypertensive drug, and statin

Figure 2 highlights the ORs for elevated hs-cTnT and NT-proBNP by blood lead levels across various subgroups. The relationship between elevated blood lead and hs-cTnT was consistent across all subgroups (all p-values for interaction > 0.05). However, the association was notably stronger for elevated NT-proBNP in Non-Hispanic Blacks (OR: 3.26 [95% CI: 2.24, 4.74], p-value for interaction < 0.008), compared to Mexican Americans (1.46 [0.99, 2.17], pairwise P-value = 0.006) and non-Hispanic Whites (1.31 [1.02, 1.68], pairwise P-value < 0.001) and Other (0.65 [0.31, 1.39], pairwise P-value < 0.001). And this association was stronger in participants with eGFR < 60 mL/min/1.73 m^2^ (OR: 2.31 [95% CI: 1.43, 3.75], p-value for interaction < 0.05), compared to individuals with eGFR ≥ 60 mL/min/1.73 m^2^ (OR: 1.44 [95% CI: 1.18, 1.75]). Figure 3 presents the ORs for elevated biomarkers by blood cadmium levels across subgroups, with no significant interactions observed (all p-values for interaction > 0.05).

### Sensitivity Analysis

Tables S4–S7 present the results of sensitivity analyses confirming the positive associations between blood lead and cadmium with hs-cTnT and NT-proBNP across different modeling conditions. These included using sex-specific quartiles for blood lead and cadmium (Table S4), using alternative cutoffs for elevated hs-cTnT and NT-proBNP (Table S5), and applying survey weights to the models (Tables S6, S7).

## Discussion

This study investigates the association between blood levels of lead and cadmium and subclinical cardiovascular disease (CVD) in U.S. adults, as indicated by the biomarkers hs-cTnT and NT-proBNP. Our findings demonstrate a significant association between elevated blood levels of these heavy metals and increased levels of hs-cTnT and NT-proBNP, suggesting a potential link between environmental exposure to lead and cadmium and early cardiovascular damage. Additionally, our study found the effect of lead on elevated NT-proBNP was more pronounced in Non-Hispanic Blacks and in those with impaired kidney function, highlighting the need for targeted interventions in these vulnerable groups.

The American Heart Association recently highlighted metals like lead and cadmium as novel, preventable CVD risk factors [2]. Our study's association between blood lead and cadmium with subclinical CVD aligns with previous research. A systematic review found that the relative risk for ischemic heart disease was 1.85 (95% CI, 1.27–2.69) when comparing the highest to the lowest third of baseline lead levels, and 1.28 (95% CI, 0.98–1.71) for cadmium [17]. Additionally, another review associated elevated blood lead levels with an increased prevalence of left ventricular hypertrophy [18]. The Strong Heart and Hortega Studies noted a positive correlation between urinary cadmium and the incidence of heart failure [19, 20]. Furthermore, exposure to lead and cadmium was linked to preclinical signs of atherosclerosis, including hypertension [21], carotid plaque formation [22], and dyslipidemia [23]. Our research further uncovered relationships between blood levels of lead and cadmium in individuals without diagnosed CVD, with cardiac biomarkers such as hs-cTnT and NT-proBNP indicating myocardial cell damage and ventricular strain. These findings underscore the significant effects of lead and cadmium exposure on early cardiomyocyte injury.

Heavy metals can induce cardiotoxicity through various mechanisms. Exposure to heavy metals can disrupt essential cardiovascular functions, including oxidative stress, chronic inflammation, impaired vascular endothelial function, and ion homeostasis disruption [8]. Specifically, lead and cadmium can directly damage cardiac cells, leading to apoptosis and the release of troponin into the bloodstream [24, 25]. Related mechanisms, such as inflammatory reactions and disrupted calcium homeostasis, can also induce cellular apoptosis. For instance, lead exposure triggers an inflammatory reaction mainly through MAP kinase activation, which plays a key role in generating pro-inflammatory cytokines (TNF-α, IL-1β, and TGF-β) [26]. Moreover, lead and cadmium interfere with calcium (Ca2 +) signaling, competing with calcium for transport via channels and pumps in the endoplasmic reticulum and interacting with calmodulin, thus disturbing calcium homeostasis and causing arrhythmia [27]. Regarding endothelial dysfunction, lead and cadmium exposure can inhibit eNOS (endothelial NO synthase) and decrease NO production, leading to increased endothelial dysfunction, vasoconstrictor activity, and vascular injury [28, 29]. Furthermore, lead and cadmium can enhance the expression of endothelial cell adhesion molecules, increase permeability, and induce oxidative stress and inflammation, ultimately promoting cardiac injury [30, 31].

We also found that the effect of blood lead on NT-proBNP was stronger in individuals with an eGFR < 60 mL/min/1.73 m^2^. Lead is primarily excreted through the kidneys, and reduced kidney function decreases the body's ability to excrete these metals, leading to higher systemic levels [32]. Additionally, environmental exposures to other heavy metals contribute to health disparities. Exposures to tungsten, uranium, cobalt, copper, and zinc have been prospectively associated with an increased risk of cardiovascular disease. The accumulation of multiple heavy metals can exacerbate kidney damage, creating a vicious cycle of toxicity [33]. These associations are further supported by a chemical-genetic-phenotype-disease network analysis, which identified pathways involving oxidative stress, cytotoxicity, and mitochondrial dysfunction [34]. Socioeconomic factors are also critical, as individuals from low-income families in poor and inner-city neighborhoods are five times more likely to have elevated blood lead levels [35]. These populations are also at greater risk for CKD development and progression due to limited access to healthcare services [36]. Moreover, individuals with chronic kidney disease (CKD) already have an increased cardiovascular risk, and lead exposure can further increase this risk. The kidneys play a significant role in clearing NT-proBNP, and reduced kidney function can lead to higher NT-proBNP levels due to decreased clearance [37]. In patients with CKD, elevations may reflect both cardiac and renal dysfunction, necessitating intensive management. the effect of blood lead on NT-proBNP was stronger in Non-Hispanic Blacks. The major sources of blood lead are residential, with lead-based paint being the most prevalent source, especially in homes built before the 1978 ban [38], and Black communities have historically been concentrated in these areas due to economic inequalities. Although blood lead levels have declined in the U.S. since the 1980s [39], racial inequities persist, with higher risk among families living in older homes, low-income families, and non-Hispanic Black households. Recent studies show that the Black population continues to have the highest average blood lead levels in the U.S. compared to non-Hispanic White or Hispanic populations [40]. Genetic susceptibility may also play a role. Divalent metal transporter-1 (DMT1), which facilitates the transport of divalent metal ions across the plasma membrane, exhibits racial and ethnic differences in expression that may contribute to disparities in blood metal levels [41, 42]. Notably, Non-Hispanic Blacks typically have lower NT-proBNP levels than non-Hispanic Whites due to genetic differences affecting NT-proBNP processing and clearance [43]. Using the same NT-proBNP cutoffs for both groups could underestimate cardiovascular risk in Non-Hispanic Blacks, suggesting that the cardiotoxicity of lead may be higher than indicated in our study. Further research is needed to clarify genetic susceptibility to lead cardiotoxicity.

The prevalence of elevated lead (≥ 3.5 µg/L) and cadmium (≥ 1.0 μg/L) was about 10% in the U.S. population, highlighting a substantial burden of metal contamination [44, 45]. Our findings provide further evidence supporting the implementation of public health strategies designed to protect individuals from such exposures. First, the prohibition of lead-based paints in children's products, such as furniture and toys in the U.S., has proven economically beneficial. For every dollar invested in controlling lead paint hazards, returns range from $17 to $221, resulting in net savings of $181–269 billion [46]. Second, Congress passed the Consumer Product Safety Improvement Act, which mandates a reduction in allowable lead content in various consumer products—such as ceramic cookware, traditional cosmetics, and jewelry—to 0.009% by weight [47]. Third, the Environmental Protection Agency (EPA) has committed to improving wastewater and drinking water infrastructure to reduce lead exposure through tap water in recent years [47]. Fourth, the EPA revised clearance levels for lead in dust from older housing, lowering the permissible amount of lead that can remain in dust on floors [48]. Lastly, the National Institute for Occupational Safety and Health (NIOSH), along with other agencies, has implemented stricter environmental and biomonitoring guidelines [49]. These measures aim not only to reduce cardiovascular disease incidence but also to address significant health disparities related to environmental injustice [50].

### Strengthens and Limitations

Our study has several strengths. First, the use of NHANES data provided a large, representative sample of the U.S. population, enabling robust statistical analysis and generalizability of findings. Second, using hs-cTnT and NT-proBNP as indicators of subclinical CVD allowed for the detection of early cardiovascular changes that precede clinical manifestations. Third, the subgroup analysis provided the clues for further personalize intervention for vulnerable population. Several limitations should be considered as well. First, the cross-sectional nature of the study limits causal inference. Longitudinal studies are needed to establish a temporal relationship between metal exposure and cardiovascular outcomes. Second, despite adjustments, residual confounding by unmeasured variables, such as diet and occupational exposure, cannot be entirely ruled out. Third, the study did not assess specific sources of lead and cadmium exposure, which could inform more targeted interventions.

## Conclusions

This study contributes to the growing evidence linking environmental lead and cadmium exposure to subclinical CVD, as indicated by elevated hs-cTnT and NT-proBNP levels. The results emphasize the importance of addressing environmental risk factors in cardiovascular prevention strategies. Future research should focus on elucidating the mechanisms involved, confirming causality, and informing policy changes to reduce heavy metal exposure and its associated health impacts.

## Supplementary Information

Below is the link to the electronic supplementary material.

Supplementary file1 (DOCX 357 KB)

## Data Availability

The datasets generated and analyzed during the current study are available on the NHANES website: https://www.cdc.gov/nchs/nhanes/index.htm

## References

[1] Roth, G. A., Mensah, G. A., Johnson, C. O., Addolorato, G., Ammirati, E., Baddour, L. M., Barengo, N. C., Beaton, A. Z., Benjamin, E. J., Benziger, C. P., Bonny, A., Brauer, M., Brodmann, M., Cahill, T. J., Carapetis, J., Catapano, A. L., Chugh, S. S., Cooper, L. T., Coresh, J., & Group, G. N. J. G. B. O. C. D. W. (2020). Global burden of cardiovascular diseases and risk factors, 1990–2019: Update from the GBD 2019 study. *Journal of the American College of Cardiology,**76*(25), 2982–3021. 10.1016/j.jacc.2020.11.010 33309175 10.1016/j.jacc.2020.11.010 PMC7755038

[2] Lamas, G. A., Bhatnagar, A., Jones, M. R., Mann, K. K., Nasir, K., Tellez-Plaza, M., Ujueta, F., & Navas-Acien, A. (2023). Contaminant metals as cardiovascular risk factors: A scientific statement from the american heart association. *Journal of the American Heart Association,**12*(13), e029852. 10.1161/jaha.123.029852 37306302 10.1161/JAHA.123.029852 PMC10356104

[3] Tchounwou, P. B., Yedjou, C. G., Patlolla, A. K., & Sutton, D. J. (2012). Heavy metal toxicity and the environment. *Exp Suppl,**101*, 133–164. 10.1007/978-3-7643-8340-4_6 22945569 10.1007/978-3-7643-8340-4_6 PMC4144270

[4] Andersson, C., Enserro, D., Sullivan, L., Wang, T. J., Januzzi, J. L., Jr., Benjamin, E. J., Vita, J. A., Hamburg, N. M., Larson, M. G., Mitchell, G. F., & Vasan, R. S. (2016). Relations of circulating GDF-15, soluble ST2, and troponin-I concentrations with vascular function in the community: The framingham heart study. *Atherosclerosis,**248*, 245–251. 10.1016/j.atherosclerosis.2016.02.013 26972631 10.1016/j.atherosclerosis.2016.02.013 PMC5018232

[5] Ndumele, C. E., Rangaswami, J., Chow, S. L., Neeland, I. J., Tuttle, K. R., Khan, S. S., Coresh, J., Mathew, R. O., Baker-Smith, C. M., Carnethon, M. R., Despres, J. P., Ho, J. E., Joseph, J. J., Kernan, W. N., Khera, A., Kosiborod, M. N., Lekavich, C. L., Lewis, E. F., Lo, K. B., & American Heart, A. (2023). Cardiovascular-kidney-metabolic health: A presidential advisory from the american heart association. *Circulation,**148*(20), 1606–1635. 10.1161/CIR.0000000000001184 37807924 10.1161/CIR.0000000000001184

[6] Liu, L., Cheng, Y. T., Xu, A., & Cheung, B. M. Y. (2023). Association between high sensitivity cardiac troponin and mortality risk in the non-diabetic population: Findings from the national health and nutrition examination survey. *Cardiovascular Diabetology*. 10.1186/s12933-023-02003-2 37904214 10.1186/s12933-023-02003-2 PMC10617237

[7] Neumann, J. T., Twerenbold, R., Weimann, J., Ballantyne, C. M., Benjamin, E. J., Costanzo, S., de Lemos, J. A., deFilippi, C. R., Di Castelnuovo, A., Donfrancesco, C., Dorr, M., Eggers, K. M., Engstrom, G., Felix, S. B., Ferrario, M. M., Gansevoort, R. T., Giampaoli, S., Giedraitis, V., Hedberg, P., & Ojeda, F. (2024). Prognostic value of cardiovascular biomarkers in the population. *JAMA,**331*(22), 1898–1909. 10.1001/jama.2024.5596 38739396 10.1001/jama.2024.5596 PMC11091824

[8] Pan, Z., Gong, T., & Liang, P. (2024). Heavy metal exposure and cardiovascular disease. *Circulation Research,**134*(9), 1160–1178. 10.1161/CIRCRESAHA.123.323617 38662861 10.1161/CIRCRESAHA.123.323617

[9] Johnson, C. L., Paulose-Ram, R., Ogden, C. L., Carroll, M. D., Kruszon-Moran, D., Dohrmann, S. M., & Curtin, L. R. (2013). National health and nutrition examination survey: Analytic guidelines, 1999–2010. *Vital Health Stat,**2*(161), 1–24. 25090154

[10] Gonzalez-Martin, R., Grau-Perez, M., Sebastian-Leon, P., Diaz-Gimeno, P., Vidal, C., Tellez-Plaza, M., & Dominguez, F. (2023). Association of blood cadmium and lead levels with self-reported reproductive lifespan and pregnancy loss: The national health and nutrition examination survey 1999–2018. *Environmental Research,**233*, 116514. 10.1016/j.envres.2023.116514 37392826 10.1016/j.envres.2023.116514

[11] Fang, M., Wang, D., Tang, O., McEvoy, J. W., Echouffo-Tcheugui, J. B., Christenson, R. H., & Selvin, E. (2023). Subclinical cardiovascular disease in US adults with and without diabetes. *Journal of the American Heart Association,**12*(11), e029083. 10.1161/JAHA.122.029083 37254959 10.1161/JAHA.122.029083 PMC10381986

[12] Medicine, T. I. F. o. C. C. a. L. *BNP, NT-proBNP, and MR-proANP Assays Analytical Characteristics Designated By Manufacturer v062024*. https://ifcc.org/ifcc-education-division/emd-committees/committee-on-clinical-applications-of-cardiac-bio-markers-c-cb/biomarkers-reference-tables/

[13] Medicine, T. I. F. o. C. C. a. L. *High Sensitivity Cardiac Troponin I and T Assay Analytical Characteristics Designated By Manufacturer v062024*. https://ifcc.org/ifcc-education-division/emd-committees/committee-on-clinical-applications-of-cardiac-bio-markers-c-cb/biomarkers-reference-tables/

[14] Levey, A. S., Bosch, J. P., Lewis, J. B., Greene, T., Rogers, N., & Roth, D. (1999). A more accurate method to estimate glomerular filtration rate from serum creatinine: A new prediction equation modification of diet in renal disease study group. *Annals of Internal Medicine,**130*(6), 461–470. 10.7326/0003-4819-130-6-199903160-00002 10075613 10.7326/0003-4819-130-6-199903160-00002

[15] McEvoy, J. W., Tang, O., Wang, D., Ndumele, C. E., Coresh, J., Christenson, R. H., & Selvin, E. (2023). Myocardial injury thresholds for 4 high-sensitivity troponin assays in U.S. adults. *Journal of the American College of Cardiology,**81*(20), 2028–2039. 37197846 10.1016/j.jacc.2023.03.403 PMC10300307

[16] *National Health and Nutrition Examination Survey tutorial*. https://wwwn.cdc.gov/nchs/nhanes/tutorials/Weighting.aspx

[17] Chowdhury, R., Ramond, A., O’Keeffe, L. M., Shahzad, S., Kunutsor, S. K., Muka, T., Gregson, J., Willeit, P., Warnakula, S., Khan, H., Chowdhury, S., Gobin, R., Franco, O. H., & Di Angelantonio, E. (2018). Environmental toxic metal contaminants and risk of cardiovascular disease: Systematic review and meta-analysis. *BMJ,**362*, k3310. 10.1136/bmj.k3310 30158148 10.1136/bmj.k3310 PMC6113772

[18] Navas-Acien, A., Guallar, E., Silbergeld, E. K., & Rothenberg, S. J. (2007). Lead exposure and cardiovascular disease—a systematic review. *Environmental Health Perspectives,**115*(3), 472–482. 10.1289/ehp.9785 17431501 10.1289/ehp.9785 PMC1849948

[19] Domingo-Relloso, A., Grau-Perez, M., Briongos-Figuero, L., Gomez-Ariza, J. L., Garcia-Barrera, T., Duenas-Laita, A., Bobb, J. F., Chaves, F. J., Kioumourtzoglou, M. A., Navas-Acien, A., Redon-Mas, J., Martin-Escudero, J. C., & Tellez-Plaza, M. (2019). The association of urine metals and metal mixtures with cardiovascular incidence in an adult population from Spain: The hortega follow-up study. *International Journal of Epidemiology,**48*(6), 1839–1849. 10.1093/ije/dyz061 31329884 10.1093/ije/dyz061 PMC6929535

[20] Tellez-Plaza, M., Guallar, E., Howard, B. V., Umans, J. G., Francesconi, K. A., Goessler, W., Silbergeld, E. K., Devereux, R. B., & Navas-Acien, A. (2013). Cadmium exposure and incident cardiovascular disease. *Epidemiology,**24*(3), 421–429. 10.1097/EDE.0b013e31828b0631 23514838 10.1097/EDE.0b013e31828b0631 PMC4142588

[21] Marques, M., Millas, I., Jimenez, A., Garcia-Colis, E., Rodriguez-Feo, J. A., Velasco, S., Barrientos, A., Casado, S., & Lopez-Farre, A. (2001). Alteration of the soluble guanylate cyclase system in the vascular wall of lead-induced hypertension in rats. *Journal of the American Society of Nephrology,**12*(12), 2594–2600. 10.1681/ASN.V12122594 11729227 10.1681/ASN.V12122594

[22] Oliveira, T. F., Batista, P. R., Leal, M. A., Campagnaro, B. P., Nogueira, B. V., Vassallo, D. V., Meyrelles, S. S., & Padilha, A. S. (2019). Chronic cadmium exposure accelerates the development of atherosclerosis and induces vascular dysfunction in the aorta of ApoE(-/-) mice. *Biological Trace Element Research,**187*(1), 163–171. 10.1007/s12011-018-1359-1 29707746 10.1007/s12011-018-1359-1

[23] Zhu, X., Fan, Y., Sheng, J., Gu, L., Tao, Q., Huang, R., Liu, K., Yang, L., Chen, G., Cao, H., Li, K., Tao, F., & Wang, S. (2021). Association between blood heavy metal concentrations and dyslipidemia in the elderly. *Biological Trace Element Research,**199*(4), 1280–1290. 10.1007/s12011-020-02270-0 32651944 10.1007/s12011-020-02270-0

[24] Chatterjee, S., Kundu, S., Sengupta, S., & Bhattacharyya, A. (2009). Divergence to apoptosis from ROS induced cell cycle arrest: Effect of cadmium. *Mutation Research,**663*(1–2), 22–31. 10.1016/j.mrfmmm.2008.12.011 19475715 10.1016/j.mrfmmm.2008.12.011

[25] Patrick, L. (2006). Lead toxicity, a review of the literature. Part 1: Exposure, evaluation, and treatment. *Alternative Medicine Review,**11*(1), 2–22. 16597190

[26] Simoes, M. R., Aguado, A., Fiorim, J., Silveira, E. A., Azevedo, B. F., Toscano, C. M., Zhenyukh, O., Briones, A. M., Alonso, M. J., Vassallo, D. V., & Salaices, M. (2015). MAPK pathway activation by chronic lead-exposure increases vascular reactivity through oxidative stress/cyclooxygenase-2-dependent pathways. *Toxicology and Applied Pharmacology,**283*(2), 127–138. 10.1016/j.taap.2015.01.005 25596430 10.1016/j.taap.2015.01.005

[27] Biagioli, M., Pifferi, S., Ragghianti, M., Bucci, S., Rizzuto, R., & Pinton, P. (2008). Endoplasmic reticulum stress and alteration in calcium homeostasis are involved in cadmium-induced apoptosis. *Cell Calcium,**43*(2), 184–195. 10.1016/j.ceca.2007.05.003 17588656 10.1016/j.ceca.2007.05.003

[28] Gonick, H. C., Ding, Y., Bondy, S. C., Ni, Z., & Vaziri, N. D. (1997). Lead-induced hypertension: Interplay of nitric oxide and reactive oxygen species. *Hypertension,**30*(6), 1487–1492. 10.1161/01.hyp.30.6.1487 9403571 10.1161/01.hyp.30.6.1487

[29] Majumder, S., Muley, A., Kolluru, G. K., Saurabh, S., Tamilarasan, K. P., Chandrasekhar, S., Reddy, H. B., Purohit, S., & Chatterjee, S. (2008). Cadmium reduces nitric oxide production by impairing phosphorylation of endothelial nitric oxide synthase. *Biochemistry and Cell Biology,**86*(1), 1–10. 10.1139/o07-146 18364740 10.1139/o07-146

[30] Camaj, P. R., Graziano, J. H., Preteni, E., Popovac, D., LoIacono, N., Balac, O., & Factor-Litvak, P. (2018). Long-term effects of environmental lead exposure on blood pressure and plasma soluble cell adhesion molecules in young adults: A follow-up study of a prospective cohort in Kosovo. *Journal of Environmental and Public Health,**2018*, 3180487. 10.1155/2018/3180487 29535789 10.1155/2018/3180487 PMC5817317

[31] Messner, B., Knoflach, M., Seubert, A., Ritsch, A., Pfaller, K., Henderson, B., Shen, Y. H., Zeller, I., Willeit, J., Laufer, G., Wick, G., Kiechl, S., & Bernhard, D. (2009). Cadmium is a novel and independent risk factor for early atherosclerosis mechanisms and in vivo relevance. *Arteriosclerosis, Thrombosis, and Vascular Biology,**29*(9), 1392–1398. 10.1161/atvbaha.109.190082 19556524 10.1161/ATVBAHA.109.190082

[32] Xu, X., Nie, S., Ding, H., & Hou, F. F. (2018). Environmental pollution and kidney diseases. *Nature Reviews. Nephrology,**14*(5), 313–324. 10.1038/nrneph.2018.11 29479079 10.1038/nrneph.2018.11

[33] Martinez-Morata, I., Schilling, K., Glabonjat, R. A., Domingo-Relloso, A., Mayer, M., McGraw, K. E., Galvez Fernandez, M., Sanchez, T. R., Nigra, A. E., Kaufman, J. D., Vaidya, D., Jones, M. R., Bancks, M. P., Barr, R. G., Shimbo, D., Post, W. S., Valeri, L., Shea, S., & Navas-Acien, A. (2024). Association of urinary metals with cardiovascular disease incidence and all-cause mortality in the multi-ethnic study of atherosclerosis (MESA). *Circulation,**150*(10), 758–769. 10.1161/CIRCULATIONAHA.124.069414 39087344 10.1161/CIRCULATIONAHA.124.069414 PMC11371385

[34] Wang, Y., Qiao, M., Yang, H., Chen, Y., Jiao, B., Liu, S., Duan, A., Wu, S., Wang, H., Yu, C., Chen, X., Duan, H., Dai, Y., & Li, B. (2024). Investigating the relationship of co-exposure to multiple metals with chronic kidney disease: An integrated perspective from epidemiology and adverse outcome pathways. *Journal of Hazardous Materials,**480*, 135844. 10.1016/j.jhazmat.2024.135844 39357351 10.1016/j.jhazmat.2024.135844

[35] Bashir, S. A. (2002). Home is where the harm is: Inadequate housing as a public health crisis. *American Journal of Public Health,**92*(5), 733–738. 10.2105/ajph.92.5.733 11988437 10.2105/ajph.92.5.733 PMC3222229

[36] Hundemer, G. L., Ravani, P., Sood, M. M., Zimmerman, D., Molnar, A. O., Moorman, D., Oliver, M. J., White, C., Hiremath, S., & Akbari, A. (2023). Social determinants of health and the transition from advanced chronic kidney disease to kidney failure. *Nephrology, Dialysis, Transplantation,**38*(7), 1682–1690. 10.1093/ndt/gfac302 36316015 10.1093/ndt/gfac302 PMC10310519

[37] Chaikijurajai, T., Demirjian, S., & Tang, W. H. W. (2023). Prognostic value of natriuretic peptide levels for adverse renal outcomes in patients with moderate to severe acute kidney injury with or without heart failure. *Journal of the American Heart Association,**12*(21), e031453. 10.1161/JAHA.123.031453 37889206 10.1161/JAHA.123.031453 PMC10727411

[38] Breysse, P. N., Cascio, W. E., Geller, A. M., Choiniere, C. J., & Ammon, M. (2022). Targeting coordinated federal efforts to address persistent hazardous exposures to lead. *American Journal of Public Health,**112*(S7), S640–S646. 10.2105/AJPH.2022.306972 36179299 10.2105/AJPH.2022.306972 PMC9528644

[39] Pirkle, J. L., Brody, D. J., Gunter, E. W., Kramer, R. A., Paschal, D. C., Flegal, K. M., & Matte, T. D. (1994). The decline in blood lead levels in the United States. The national health and nutrition examination surveys (NHANES). *JAMA,**272*(4), 284–291. 8028141

[40] Theppeang, K., Glass, T. A., Bandeen-Roche, K., Todd, A. C., Rohde, C. A., & Schwartz, B. S. (2008). Gender and race/ethnicity differences in lead dose biomarkers. *American Journal of Public Health,**98*(7), 1248–1255. 10.2105/AJPH.2007.118505 18511728 10.2105/AJPH.2007.118505 PMC2424096

[41] Ettinger, A. S., Bovet, P., Plange-Rhule, J., Forrester, T. E., Lambert, E. V., Lupoli, N., Shine, J., Dugas, L. R., Shoham, D., Durazo-Arvizu, R. A., Cooper, R. S., & Luke, A. (2014). Distribution of metals exposure and associations with cardiometabolic risk factors in the “modeling the epidemiologic transition study.” *Environmental Health,**13*, 90. 10.1186/1476-069X-13-90 25374160 10.1186/1476-069X-13-90 PMC4240881

[42] Ngueta, G. (2014). Racial disparities in children’s blood lead levels: Possible implication of divalent metal transporter 1. *Medical Hypotheses,**82*(1), 71–73. 10.1016/j.mehy.2013.11.008 24290248 10.1016/j.mehy.2013.11.008

[43] Patel, N., Russell, G. K., Musunuru, K., Gutierrez, O. M., Halade, G., Kain, V., Lv, W., Prabhu, S. D., Margulies, K. B., Cappola, T. P., Arora, G., Wang, T. J., & Arora, P. (2019). Race, natriuretic peptides, and high-carbohydrate challenge: A clinical trial. *Circulation Research,**125*(11), 957–968. 10.1161/CIRCRESAHA.119.315026 31588864 10.1161/CIRCRESAHA.119.315026 PMC7033629

[44] Shibeeb, S., Abdallah, A., & Shi, Z. (2024). Blood homocysteine levels mediate the association between blood lead levels and cardiovascular mortality. *Cardiovascular Toxicology,**24*(1), 62–70. 10.1007/s12012-023-09819-0 38231351 10.1007/s12012-023-09819-0 PMC10838245

[45] Wen, X., Li, T., & Xu, X. (2022). Cadmium exposure in US adults, research based on the national health and nutrition examination survey from 1988 to 2018. *Environmental Science and Pollution Research International,**29*(15), 22293–22305. 10.1007/s11356-021-17484-5 34782977 10.1007/s11356-021-17484-5

[46] Gould, E. (2009). Childhood lead poisoning: Conservative estimates of the social and economic benefits of lead hazard control. *Environmental Health Perspectives,**117*(7), 1162–1167. 10.1289/ehp.0800408 19654928 10.1289/ehp.0800408 PMC2717145

[47] Dignam, T., Kaufmann, R. B., LeStourgeon, L., & Brown, M. J. (2019). Control of lead sources in the United States, 1970–2017: Public Health Progress and Current Challenges to Eliminating Lead Exposure. *J Public Health Manag Pract*, *25 Suppl 1, Lead Poisoning Prevention *(Suppl 1 LEAD POISONING PREVENTION), S13-S22. 10.1097/PHH.0000000000000889 10.1097/PHH.0000000000000889 PMC6522252 30507765

[48] Hazard Standards and Clearance Levels for Lead in Paint, Dust and Soil (TSCA Sections 402 and 403). https://www.epa.gov/lead/hazard-standards-and-clearance-levels-lead-paint-dust-and-soil-tsca-sections-402-and-403

[49] OECD. Occupational Biomonitoring Guidance Document. OECD Series on Testing and Assessment, no. 370, 2022, OECD Publishing, 10.1787/11bc2c7a-en. In.

[50] Sampson, R. J., & Winter, A. S. (2016). The racial ecology of lead poisoning: Toxic inequality in chicago neighborhoods, 1995–2013. *Du Bois Review: Social Science Research on Race,**13*(2), 261–283. 10.1017/S1742058X16000151

