# Supplementary material for: Associations Between Lead and Cadmium Exposure and Subclinical Cardiovascular Disease in U.S. Adults
Source: Cardiovasc Toxicol. 2025 Jan 28;25(2):282–93. doi: 10.1007/s12012-024-09955-1 (PMC11811258; doi:10.1007/s12012-024-09955-1)
Supplement: Supplementary file 1 — Supplementary file1 (DOCX 357 KB) [file 12012_2024_9955_MOESM1_ESM.docx]

Supplementary materials

Figure S1. Flow chart of study population

Figure S2. Correlations of blood lead and cadmium with Hs-cTnT and NT-proBNP

Table S1. Range of Blood Lead and Cadmium concentration by quartiles

Table S2. Baseline characteristic according to blood Lead quartiles

Table S3. Baseline characteristic according to blood Cadmium quartiles

Table S4. Sensitivity analysis: odd ratio of elevated Hs-cTnT and NT-proBNP by sex-specific quartiles blood Lead and Cadmium

Table S5. Sensitivity analysis: odd ratio of elevated Hs-cTnT and NT-proBNP using different cutoffs for Hs-cTnT and NT-proBNP

Table S6. Sensitivity analysis: odd ratio of elevated Hs-cTnT and NT-proBNP by blood Lead and Cadmium for complex survey using MEC exam weight

Table S7. Sensitivity analysis: odd ratio of elevated Hs-cTnT and NT-proBNP by blood Lead and Cadmium for complex survey using Hs-cTnT and NT-proBNP measurement weight


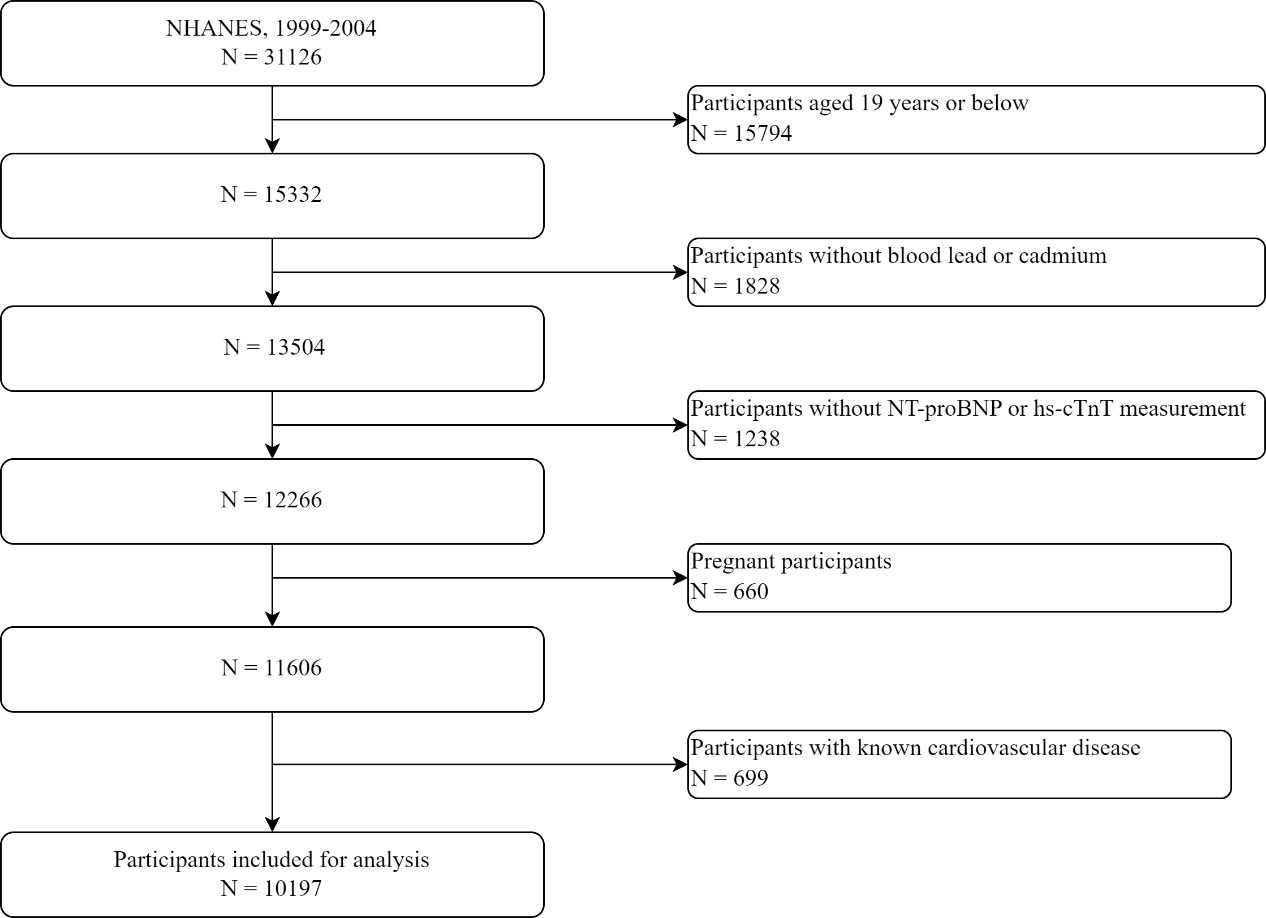


Figure S1. Flow chart of study population. NHANES, National Health and Nutrition Examination Survey. hs-cTnT, high sensitivity cardiac troponin, NT-proBNP, N-terminal pro b-type natriuretic peptide.


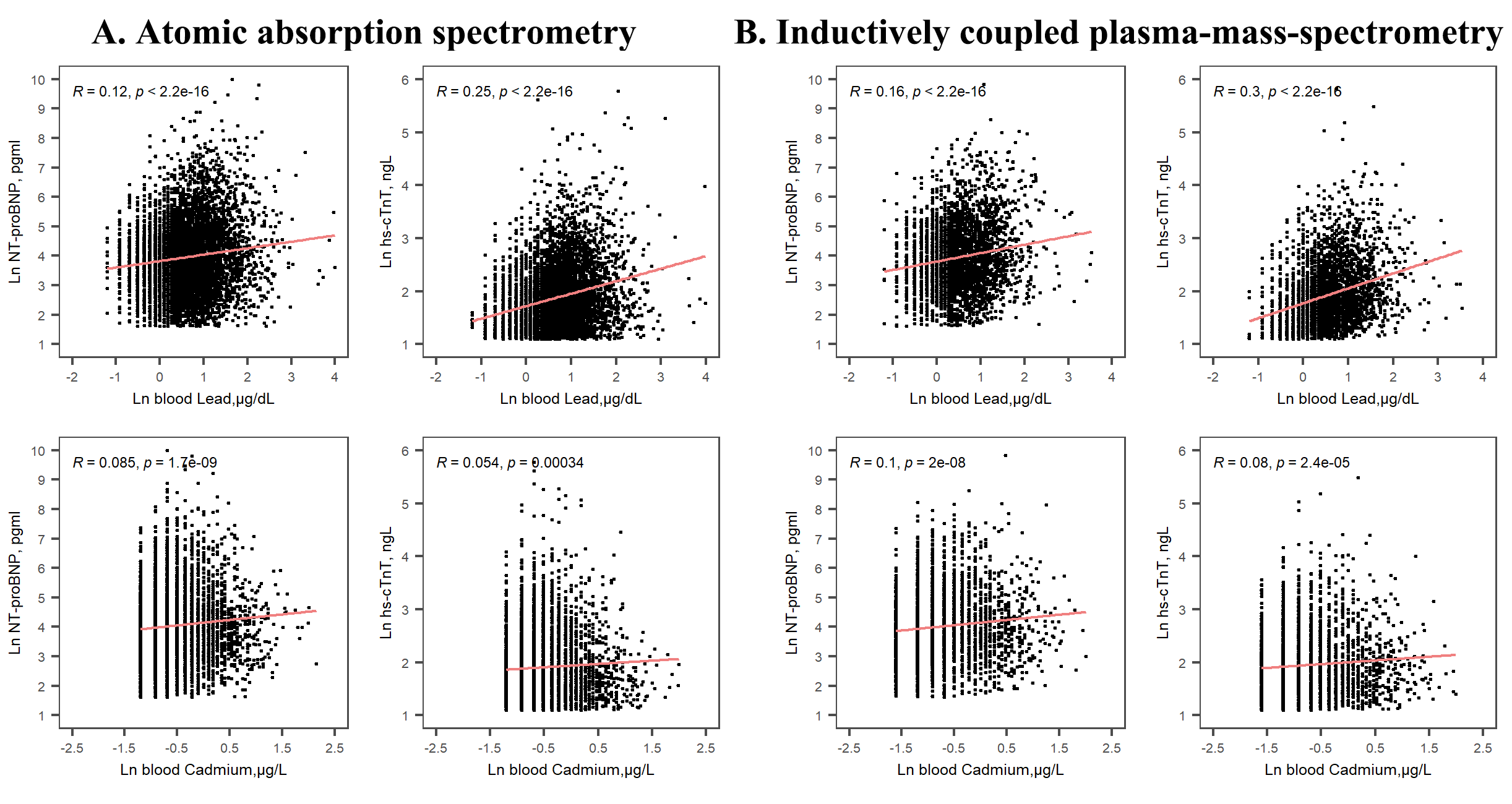


Figure S2. Correlations of blood lead and cadmium with Hs-cTnT and NT-proBNP.

The Pearson correlation coefficient (R) and p-value were shown

Ln, log-transformed, hs-cTnT, high sensitivity cardiac troponin, NT-proBNP, N-terminal pro b-type natriuretic peptide.

Table S1. Range of Blood Lead and Cadmium concentration by quartiles

| Overall quartiles | | | | | |
| --- | --- | --- | --- | --- | --- |
|  |  | Q1 | Q2 | Q3 | Q4 |
| NHANES, 1999-2002 | Blood lead, μg/dL | < 1.2 | 1.2 - 1.8 | 1.8 - 2.7 | ≥ 2.7 |
|  | Blood cadmium, μg/L | < 0.3 | 0.3 - 0.4 | 0.4 - 0.7 | ≥ 0.7 |
| NHANES, 2003-2004 | Blood lead, μg/dL | < 1.1 | 1.1 - 1.6 | 1.6 - 2.5 | ≥ 2.5 |
|  | Blood cadmium, μg/L | < 0.2 | 0.2 - 0.4 | 0.4 - 0.6 | ≥ 0.6 |
| Sex-specific quartiles | | | | | |
|  | | **Male** | | | |
| NHANES, 1999-2002 | Blood lead, μg/dL | < 1.4 | 1.4 – 2.1 | 2.1 – 3.2 | ≥ 3.2 |
|  | Blood cadmium, μg/L | < 0.2 | 0.2 - 0.4 | 0.4 - 0.7 | ≥ 0.7 |
| NHANES, 2003-2004 | Blood lead, μg/dL | < 1.3 | 1.3 - 1.9 | 1.9 - 2.9 | ≥ 2.9 |
|  | Blood cadmium, μg/L | < 0.2 | 0.2 - 0.3 | 0.3 - 0.6 | ≥ 0.6 |
|  |  | **Female** | | | |
| NHANES, 1999-2002 | Blood lead, μg/dL | < 0.9 | 0.9 - 1.4 | 1.4 - 2.1 | ≥ 2.1 |
|  | Blood cadmium, μg/L | < 0.3 | 0.3 - 0.4 | 0.4 - 0.7 | ≥ 0.7 |
| NHANES, 2003-2004 | Blood lead, μg/dL | < 0.9 | 0.9 - 1.4 | 1.4 - 2.1 | ≥ 2.1 |
|  | Blood cadmium, μg/L | < 0.3 | 0.3 - 0.4 | 0.4 - 0.7 | ≥ 0.7 |

NHANES, National Health and Nutrition Examination Survey.

Atomic absorption spectrometry was used in NHANES,1999-2002, inductively coupled plasma mass spectrometry was used in NHANES,2003-2004.

Table S2. Baseline characteristic according to blood Lead quartiles

|  |  | Blood Lead, μg/dL | | | | |
| --- | --- | --- | --- | --- | --- | --- |
|  | Overall | Q1 | Q2 | Q3 | Q4 | P-value |
|  | 10197 | 2550 | 2549 | 2549 | 2549 |  |
| Age (years) | 48.8 ± 18.2 | 39.6 ± 16.3 | 47.0 ± 17.4 | 52.7 ± 17.3 | 55.8 ± 17.4 | <0.001 |
| Gender, % |  |  |  |  |  | <0.001 |
| Male | 5063 (49.7) | 711 (27.9) | 1130 (44.3) | 1450 (56.9) | 1772 (69.5) |  |
| Female | 5134 (50.3) | 1839 (72.1) | 1419 (55.7) | 1099 (43.1) | 777 (30.5) |  |
| Race/Ethnicity, % |  |  |  |  |  | <0.001 |
| Mexican-American | 2367 (23.2) | 549 (21.5) | 563 (22.1) | 581 (22.8) | 674 (26.4) |  |
| Non-Hispanic White | 5115 (50.2) | 1367 (53.6) | 1318 (51.7) | 1299 (51.0) | 1131 (44.4) |  |
| Non-Hispanic Black | 1876 (18.4) | 408 (16.0) | 444 (17.4) | 465 (18.2) | 559 (21.9) |  |
| Other | 839 (8.2) | 226 (8.9) | 224 (8.8) | 204 (8.0) | 185 (7.3) |  |
| Education, % |  |  |  |  |  | <0.001 |
| Less than high school | 3161 (31.0) | 511 (20.0) | 711 (27.9) | 805 (31.6) | 1134 (44.5) |  |
| High school | 2451 (24.0) | 613 (24.0) | 577 (22.6) | 663 (26.0) | 598 (23.5) |  |
| College or higher | 4585 (45.0) | 1426 (55.9) | 1261 (49.5) | 1081 (42.4) | 817 (32.1) |  |
| Smoking status, % |  |  |  |  |  | <0.001 |
| Current smoker | 2656 (26.0) | 366 (14.4) | 609 (23.9) | 748 (29.3) | 933 (36.6) |  |
| Former smoker | 2593 (25.4) | 446 (17.5) | 645 (25.3) | 715 (28.1) | 787 (30.9) |  |
| Nonsmoker | 4948 (48.5) | 1738 (68.2) | 1295 (50.8) | 1086 (42.6) | 829 (32.5) |  |
| SBP (mmHg) | 126.3 ± 20.2 | 120.0 ± 17.6 | 124.7 ± 18.9 | 128.8 ± 20.8 | 131.4 ± 21.4 | <0.001 |
| DBP (mmHg) | 71.4 ± 13.1 | 70.2 ± 11.7 | 71.3 ± 12.3 | 72.4 ± 13.4 | 71.8 ± 14.6 | <0.001 |
| BMI (kg/m2) | 28.2 ± 6.1 | 28.6 ± 6.9 | 28.5 ± 6.2 | 28.3 ± 5.7 | 27.3 ± 5.1 | <0.001 |
| TC (mg/dL) | 202.8 ± 41.4 | 194.3 ± 39.2 | 201.9 ± 42.9 | 207.8 ± 41.1 | 207.1 ± 40.7 | <0.001 |
| HbA1c (%) | 5.5 ± 1.0 | 5.4 ± 0.9 | 5.5 ± 1.0 | 5.6 ± 1.0 | 5.5 ± 0.9 | <0.001 |
| eGFR (mL/min/1.73 m2) | 92.8 ± 31.4 | 100.5 ± 39.5 | 93.2 ± 27.3 | 88.7 ± 26.1 | 88.6 ± 29.1 | <0.001 |
| NT-proBNP (pg/mL) | 45.6 [21.9, 98.6] | 41.0 [20.8, 79.0] | 43.84 [21.4, 92.9] | 45.5 [21.5, 102.3] | 54.8 [23.6, 141.5] | <0.001 |
| Hs-cTnT (ng/L) | 5.3 [3.7, 8.2] | 4.0 [3.1, 5.8] | 5.08 [3.6, 7.4] | 6.0 [4.1, 9.4] | 6.8 [4.7, 11.2] | <0.001 |
| Hypertension, % | 3146 (30.9) | 601 (23.6) | 763 (29.9) | 857 (33.6) | 925 (36.3) | <0.001 |
| Anti-hypertensive drug, % | 1854 (18.2) | 327 (12.8) | 462 (18.1) | 503 (19.7) | 562 (22.0) | <0.001 |
| Diabetes mellitus, % | 1082 (10.6) | 248 (9.7) | 298 (11.7) | 276 (10.8) | 260 (10.2) | <0.001 |
| Anti-diabetic drug, % | 662 (6.5) | 175 (6.9) | 189 (7.4) | 177 (6.9) | 121 (4.7) | <0.001 |
| Statins, % | 757 (7.4) | 125 (4.9) | 195 (7.7) | 251 (9.8) | 186 (7.3) | <0.001 |
| Elevated Hs-cTnT, % | 1978 (19.4) | 313 (12.3) | 445 (17.5) | 530 (20.8) | 690 (27.1) | <0.001 |
| Elevated NT-proBNP, % | 544 (5.3) | 48 (1.9) | 92 (3.6) | 142 (5.6) | 262 (10.3) | <0.001 |

Continuous variables are expressed as mean ± standard deviation or as median [IQR].

Categorical variables are expressed as number (percent)

Elevated NT-proBNP was defined as blood NT-proBNP ≥ 125 pg/ml. Elevated hs-cTnT was defined as blood hs-cTnT ≥ 19ng/L

SBP, systolic blood pressure, DBP, diastolic blood pressure, BMI, body mass index, TC, total cholesterol, HbA1c, hemoglobin A1c, eGFR, estimated glomerular rate, Hs-cTnT, high sensitivity cardiac troponin, NT-proBNP, N-terminal pro b-type natriuretic peptide

Table S3. Baseline characteristic according to blood Cadmium quartiles

|  |  | Blood Cadmium, μg/L | | | | |
| --- | --- | --- | --- | --- | --- | --- |
|  | Overall | Q1 | Q2 | Q3 | Q4 | P-value |
|  | 10197 | 2550 | 2549 | 2549 | 2549 |  |
| Age (years) | 48.8 ± 18.2 | 41.7 ± 16.4 | 48.2 ± 17.8 | 54.4 ± 18.4 | 50.7 ± 17.7 | <0.001 |
| Gender, % |  |  |  |  |  |  |
| Male | 5063 (49.7) | 1474 (57.8) | 1215 (47.7) | 1113 (43.7) | 1261 (49.5) |  |
| Female | 5134 (50.3) | 1076 (42.2) | 1334 (52.3) | 1436 (56.3) | 1288 (50.5) |  |
| Race/Ethnicity, % |  |  |  |  |  | <0.001 |
| Mexican-American | 2367 (23.2) | 578 (22.7) | 682 (26.8) | 662 (26.0) | 445 (17.5) |  |
| Non-Hispanic White | 5115 (50.2) | 1300 (51.0) | 1233 (48.4) | 1255 (49.2) | 1327 (52.1) |  |
| Non-Hispanic Black | 1876 (18.4) | 494 (19.4) | 407 (16.0) | 416 (16.3) | 559 (21.9) |  |
| Other | 839 (8.2) | 178 (7.0) | 227 (8.9) | 216 (8.5) | 218 (8.6) |  |
| Education, % |  |  |  |  |  | <0.001 |
| Less than high school | 3161 (31.0) | 590 (23.1) | 753 (29.5) | 848 (33.3) | 970 (38.1) |  |
| High school | 2451 (24.0) | 570 (22.4) | 554 (21.7) | 622 (24.4) | 705 (27.7) |  |
| College or higher | 4585 (45.0) | 1390 (54.5) | 1242 (48.7) | 1079 (42.3) | 874 (34.3) |  |
| Smoking status, % |  |  |  |  |  | <0.001 |
| Current smoker | 2656 (26.0) | 180 (7.1) | 257 (10.1) | 526 (20.6) | 1693 (66.4) |  |
| Former smoker | 2593 (25.4) | 566 (22.2) | 731 (28.7) | 857 (33.6) | 439 (17.2) |  |
| Nonsmoker | 4948 (48.5) | 1804 (70.7) | 1561 (61.2) | 1166 (45.7) | 417 (16.4) |  |
| SBP (mmHg) | 126.3 ± 20.2 | 121.9 ± 17.4 | 125.7 ± 19.8 | 129.5 ± 21.6 | 127.9 ± 20.8 | <0.001 |
| DBP (mmHg) | 71.4 ± 13.1 | 71.6 ± 11.7 | 71.9 ± 12.3 | 71.0 ± 14.1 | 71.2 ± 13.9 | 0.097 |
| BMI (kg/m2) | 28.2 ± 6.1 | 28.7 ± 6.5 | 28.5 ± 5.9 | 28.4 ± 6.0 | 27.1 ± 5.7 | <0.001 |
| TC (mg/dL) | 202.8 ± 41.4 | 198.3 ± 41.8 | 202.1 ± 39.5 | 206.4 ± 42.2 | 204.3 ± 41.4 | <0.001 |
| HbA1c (%) | 5.5 ± 1.0 | 5.5 ± 1.0 | 5.5 ± 1.0 | 5.6 ± 1.0 | 5.5 ± 0.9 | <0.001 |
| eGFR (mL/min/1.73 m2) | 92.8 ± 31.4 | 95.9 ± 37.9 | 94.5 ± 29.7 | 89.7 ± 28.7 | 90.9 ± 27.7 | <0.001 |
| NT-proBNP (pg/mL) | 45.6 [21.9, 98.6] | 33.4 [16.2, 67.1] | 43.4 [21.2, 88.5] | 57.4 [26.2, 132.6] | 53.9 [25.1, 119.7] | <0.001 |
| Hs-cTnT (ng/L) | 5.3 [3.7, 8.2] | 5.0 [3.6, 7.1] | 5.1 [3.6, 7.7] | 5.8 [3.9, 9.7] | 5.6 [3.9, 9.1] | <0.001 |
| Hypertension, % | 3146 (30.9) | 631 (24.7) | 751 (29.5) | 940 (36.9) | 824 (32.3) | <0.001 |
| Anti-hypertensive drug, % | 1854 (18.2) | 360 (14.1) | 438 (17.2) | 577 (22.6) | 479 (18.8) | <0.001 |
| Diabetes mellitus, % | 1082 (10.6) | 263 (10.3) | 270 (10.6) | 303 (11.9) | 246 (9.7) | 0.07 |
| Anti-diabetic drug, % | 662 (6.5) | 170 (6.7) | 161 (6.3) | 197 (7.7) | 134 (5.3) | 0.004 |
| Statins, % | 757 (7.4) | 141 (5.5) | 212 (8.3) | 235 (9.2) | 169 (6.6) | <0.001 |
| Elevated Hs-cTnT, % | 1978 (19.4) | 254 (10.0) | 436 (17.1) | 669 (26.2) | 619 (24.3) | <0.001 |
| Elevated NT-proBNP, % | 544 (5.3) | 82 (3.2) | 99 (3.9) | 181 (7.1) | 182 (7.1) | <0.001 |

Continuous variables are expressed as mean ± standard deviation or as median [IQR].

Categorical variables are expressed as number (percent)

Elevated NT-proBNP was defined as blood NT-proBNP ≥ 125 pg/ml. Elevated hs-cTnT was defined as blood hs-cTnT ≥ 19ng/L

SBP, systolic blood pressure, DBP, diastolic blood pressure, BMI, body mass index, TC, total cholesterol, HbA1c, hemoglobin A1c, eGFR, estimated glomerular rate, Hs-cTnT, high sensitivity cardiac troponin, NT-proBNP, N-terminal pro b-type natriuretic peptide

Table S4. Odd ratios (95% Confidence interval) of elevated Hs-cTnT and NT-proBNP by Sex-specific Quartiles Blood Lead and Cadmium

|  | Elevated Hs-cTnT (N = 544) | | | Elevated NT-proBNP (N = 1978) | | |
| --- | --- | --- | --- | --- | --- | --- |
|  | Cases/total | Model 1 | Model 2 | Cases/total | Model 1 | Model 2 |
| Blood Lead |  |  |  |  |  |  |
| Quartile 1 | 63/2551 | Ref | Ref | 260/2551 | Ref | Ref |
| Quartile 2 | 91/2549 | 1.46 (1.06, 2.03) | 0.79 (0.55, 1.14) | 388/2549 | 1.58 (1.34, 1.87) | 0.84 (0.68, 1.03) |
| Quartile 3 | 144/2549 | 2.36 (1.75, 3.20) | 1.01 (0.72, 1.42) | 537/2549 | 2.35 (2.00, 2.76) | 0.92 (0.75, 1.12) |
| Quartile 4 | 246/2548 | 4.22 (3.18, 5.60) | 1.29 (0.93, 1.80) | 793/2548 | 3.98 (3.42, 4.64) | 1.13 (0.93, 1.37) |
| Blood Cadmium |  |  |  |  |  |  |
| Quartile 1 | 84/2551 | Ref | Ref | 281/2551 | Ref | Ref |
| Quartile 2 | 91/2549 | 1.09 (0.80, 1.47) | 0.70 (0.50, 0.98) | 432/2549 | 1.65 (1.40, 1.94) | 1.07 (0.88, 1.30) |
| Quartile 3 | 187/2549 | 2.33 (1.79, 3.03) | 0.96 (0.71, 1.29) | 647/2549 | 2.75 (2.36, 3.20) | 1.21 (1.00, 1.47) |
| Quartile 4 | 182/2548 | 2.26 (1.73, 2.94) | 1.38 (1.02, 1.87) | 618/2548 | 2.59 (2.22, 3.02) | 1.56 (1.29, 1.89) |

Hs-cTnT, high sensitivity cardiac troponin, NT-proBNP, N-terminal pro b-type natriuretic peptide

Elevated NT-proBNP was defined as blood NT-proBNP ≥ 125 pg/ml. Elevated hs-cTnT was defined as blood hs-cTnT ≥ 19ng/L.

Model 1, crude model.

Model 2, adjusted for age, gender, race/ethnicity, smoking status, systolic blood pressure, body mass index, total cholesterol, hemoglobin A1c, estimated glomerular rate, diabetes, anti-diabetic drug, hypertension, anti-hypertensive drug, and statin.

Table S5. Sensitivity analysis: odd ratios (95% Confidence interval) of elevated Hs-cTnT and NT-proBNP using Different Cutoffs for Hs-cTnT and NT-proBNP

|  | Elevated Hs-cTnT (N = 802) | | | Elevated NT-proBNP (N = 1471) | | |
| --- | --- | --- | --- | --- | --- | --- |
|  | Cases/total | Model 1 | Model 2 | Cases/total | Model 1 | Model 2 |
| Blood Lead |  |  |  |  |  |  |
| Quartile 1 | 67/2550 | Ref | Ref | 257/2550 | Ref | Ref |
| Quartile 2 | 133/2549 | 2.04 (1.51, 2.75) | 0.88 (0.62, 1.25) | 347/2549 | 1.41 (1.18, 1.67) | 1.13 (0.93, 1.36) |
| Quartile 3 | 213/2549 | 3.38 (2.55, 4.47) | 0.84 (0.60, 1.18) | 368/2549 | 1.51 (1.27, 1.78) | 1.03 (0.85, 1.25) |
| Quartile 4 | 389/2549 | 6.67 (5.12, 8.70) | 1.18 (0.85, 1.65) | 499/2549 | 2.17 (1.85, 2.55) | 1.54 (1.26, 1.87) |
| Not elevated | 581/8900 | Ref | Ref | 1178/8900 | Ref | Ref |
| Elevated | 221/1297 | 2.94 (2.49, 3.48) | 1.27 (1.03, 1.58) | 293/1297 | 1.91 (1.66, 2.21) | 1.77 (1.50, 2.10) |
| Blood Cadmium |  |  |  |  |  |  |
| Quartile 1 | 132/2550 | Ref | Ref | 208/2550 | Ref | Ref |
| Quartile 2 | 176/2549 | 1.36 (1.08, 1.71) | 1.05 (0.79, 1.39) | 320/2549 | 1.62 (1.34, 1.94) | 1.12 (0.92, 1.37) |
| Quartile 3 | 251/2549 | 2.00 (1.61, 2.49) | 1.09 (0.83, 1.42) | 461/2549 | 2.49 (2.09, 2.96) | 1.25 (1.03, 1.52) |
| Quartile 4 | 243/2549 | 1.93 (1.55, 2.40) | 1.46 (1.12, 1.91) | 482/2549 | 2.63 (2.21, 3.12) | 1.67 (1.38, 2.02) |
| Not elevated | 665/8623 | Ref | Ref | 1190/8623 | Ref | Ref |
| Elevated | 137/1574 | 1.14 (0.94, 1.38) | 1.34 (1.06, 1.70) | 281/1574 | 1.36 (1.18, 1.57) | 1.52 (1.3, 1.79) |

Hs-cTnT, high sensitivity cardiac troponin, NT-proBNP, N-terminal pro b-type natriuretic peptide

Elevated blood lead was defined as blood lead concentration > 3.5 μg/dL for inductively coupled plasma mass spectrometry and > 3.8 μg/dL for atomic absorption spectrometry.

Elevated blood cadmium was defined as blood cadmium concentration > 1.0 μg/L for inductively coupled plasma mass spectrometry and > 0.9 μg/L for atomic absorption spectrometry.

**Elevated NT-proBNP was defined as blood NT-proBNP ≥125 pg/mL for adults aged <75 years and ≥450 pg/mL for adults aged ≥75 years**

**Elevated hs-cTnT was defined as blood hs-cTnT ≥ 14 ng/L for women and ≥ 22 ng/L for men.**

Model 1, crude model.

Model 2, adjusted for age, gender, race/ethnicity, smoking status, systolic blood pressure, body mass index, total cholesterol, hemoglobin A1c, estimated glomerular rate, diabetes, anti-diabetic drug, hypertension, anti-hypertensive drug, and statin.

Table S6. Odd ratios (95% Confidence interval) of Elevated Hs-cTnT and NT-proBNP by Blood Lead and Cadmium for Complex Survey **using mobile examination center exam weight**

|  | Elevated Hs-cTnT (N = 544) | | | Elevated NT-proBNP (N = 1978) | | |
| --- | --- | --- | --- | --- | --- | --- |
|  | Cases/total | Model 1 | Model 2 | Cases/total | Model 1 | Model 2 |
| Blood Lead |  |  |  |  |  |  |
| Quartile 1 | 48/2550 | Ref | Ref | 313/2550 | Ref | Ref |
| Quartile 2 | 92/2549 | 1.38 (0.99, 1.93) | 0.66 (0.45, 0.96) | 445/2549 | 1.35 (1.08, 1.69) | 0.91 (0.72, 1.15) |
| Quartile 3 | 142/2549 | 2.18 (1.42, 3.34) | 0.67 (0.43, 1.05) | 530/2549 | 1.53 (1.22, 1.92) | 0.80 (0.62, 1.04) |
| Quartile 4 | 262/2549 | 4.60 (3.24, 6.53) | 1.00 (0.65, 1.52) | 690/2549 | 2.11 (1.74, 2.57) | 1.03 (0.81, 1.32) |
| Not elevated | 392/8900 | Ref | Ref | 1580/8900 | Ref | Ref |
| Elevated | 152/1297 | 2.99 (2.37, 3.76) | 1.36 (1.02, 1.80) | 398/1297 | 1.82 (1.51, 2.20) | 1.41 (1.14, 1.76) |
| Blood Cadmium |  |  |  |  |  |  |
| Quartile 1 | 82/2550 | Ref | Ref | 254/2550 | Ref | Ref |
| Quartile 2 | 99/2549 | 0.95 (0.60, 1.51) | 0.62 (0.38, 1.02) | 436/2549 | 1.92 (1.54, 2.38) | 1.13 (0.88, 1.45) |
| Quartile 3 | 181/2549 | 1.86 (1.23, 2.83) | 0.83 (0.55, 1.25) | 669/2549 | 3.21 (2.63, 3.91) | 1.25 (0.99, 1.57) |
| Quartile 4 | 182/2549 | 1.86 (1.24, 2.79) | 1.27 (0.78, 2.07) | 619/2549 | 2.76 (2.25, 3.38) | 1.44 (1.06, 1.96) |
| Not elevated | 452/8623 | Ref | Ref | 1638/8623 | Ref | Ref |
| Elevated | 92/1574 | 1.21 (0.92, 1.59) | 1.54 (1.05, 2.26) | 340/1574 | 1.17 (1.03, 1.33) | 1.21 (1.02, 1.50) |

Hs-cTnT, high sensitivity cardiac troponin, NT-proBNP, N-terminal pro b-type natriuretic peptide

Elevated blood lead was defined as blood lead concentration > 3.5 μg/dL for inductively coupled plasma mass spectrometry and > 3.8 μg/dL for atomic absorption spectrometry.

Elevated blood cadmium was defined as blood cadmium concentration > 1.0 μg/L for inductively coupled plasma mass spectrometry and > 0.9 μg/L for atomic absorption spectrometry.

Elevated NT-proBNP was defined as blood NT-proBNP ≥ 125 pg/ml. Elevated hs-cTnT was defined as blood hs-cTnT ≥ 19ng/L.

Model 1, crude model.

Model 2, adjusted for age, gender, race/ethnicity, smoking status, systolic blood pressure, body mass index, total cholesterol, hemoglobin A1c, estimated glomerular rate, diabetes, anti-diabetic drug, hypertension, anti-hypertensive drug, and statin.

Table S7. Odd ratio of elevated Hs-cTnT and NT-proBNP by blood Lead and Cadmium for complex survey **using Hs-cTnT and NT-proBNP measurement weight**

|  | Elevated Hs-cTnT (N = 544) | | | Elevated NT-proBNP (N = 1978) | | |
| --- | --- | --- | --- | --- | --- | --- |
|  | Cases/total | Model 1 | Model 2 | Cases/total | Model 1 | Model 2 |
| Blood Lead |  |  |  |  |  |  |
| Quartile 1 | 48/2550 | Ref | Ref | 313/2550 | Ref | Ref |
| Quartile 2 | 92/2549 | 1.40 (1.01, 1.94) | 0.67 (0.46, 0.97) | 445/2549 | 1.36 (1.09, 1.70) | 0.91 (0.72, 1.15) |
| Quartile 3 | 142/2549 | 2.19 (1.43, 3.35) | 0.67 (0.43, 1.05) | 530/2549 | 1.54 (1.23, 1.92) | 0.80 (0.62, 1.04) |
| Quartile 4 | 262/2549 | 4.68 (3.32, 6.59) | 1.02 (0.67, 1.54) | 690/2549 | 2.13 (1.76, 2.59) | 1.03 (0.81, 1.32) |
| Not elevated | 392/8900 | Ref | Ref | 1580/8900 | Ref | Ref |
| Elevated | 152/1297 | 3.02 (2.40, 3.79) | 1.37 (1.03, 1.81) | 398/1297 | 1.84 (1.53, 2.22) | 1.43 (1.15, 1.77) |
| Blood Cadmium |  |  |  |  |  |  |
| Quartile 1 | 82/2550 | Ref | Ref | 254/2550 | Ref | Ref |
| Quartile 2 | 99/2549 | 0.94 (0.60, 1.48) | 0.62 (0.38, 1.02) | 436/2549 | 1.91 (1.53, 2.37) | 1.13 (0.88, 1.45) |
| Quartile 3 | 181/2549 | 1.85 (1.23, 2.78) | 0.83 (0.55, 1.25) | 669/2549 | 3.19 (2.62, 3.89) | 1.25 (0.99, 1.57) |
| Quartile 4 | 182/2549 | 1.84 (1.23, 2.75) | 1.27 (0.78, 2.07) | 619/2549 | 2.76 (2.25, 3.37) | 1.45 (1.06, 1.96) |
| Not elevated | 452/8623 | Ref | Ref | 1638/8623 | Ref | Ref |
| Elevated | 92/1574 | 1.20 (0.91, 1.58) | 1.52 (1.04, 2.23) | 340/1574 | 1.17 (1.02, 1.34) | 1.15 (0.92, 1.44) |

The weights for Hs-cTnT and NT-proBNP measurement are the same.

Hs-cTnT, high sensitivity cardiac troponin, NT-proBNP, N-terminal pro b-type natriuretic peptide

Elevated blood lead was defined as blood lead concentration > 3.5 μg/dL for inductively coupled plasma mass spectrometry and > 3.8 μg/dL for atomic absorption spectrometry.

Elevated blood cadmium was defined as blood cadmium concentration > 1.0 μg/L for inductively coupled plasma mass spectrometry and > 0.9 μg/L for atomic absorption spectrometry.

Elevated NT-proBNP was defined as blood NT-proBNP ≥ 125 pg/ml. Elevated hs-cTnT was defined as blood hs-cTnT ≥ 19ng/L.

Model 1, crude model.

Model 2, adjusted for age, gender, race/ethnicity, smoking status, systolic blood pressure, body mass index, total cholesterol, hemoglobin A1c, estimated glomerular rate, diabetes, anti-diabetic drug, hypertension, anti-hypertensive drug, and statin.
